# Rapid and sensitive detection of uranyl ion with citrate-stabilized silver nanoparticles by the surface-enhanced Raman scattering technique

**DOI:** 10.1098/rsos.181099

**Published:** 2018-11-28

**Authors:** Jiaolai Jiang, Shaofei Wang, Hui Deng, Haoxi Wu, Jun Chen, Junsheng Liao

**Affiliations:** Institute of Materials, China Academy of Engineering Physics, PO Box No. 9-11, Mianyang, Sichuan 621907, People's Republic of China

**Keywords:** silver nanoparticles, citrate, uranyl ion detection, quantification, internal reference, surface-enhanced Raman scattering

## Abstract

Uranium contamination poses a huge threat to human health due to its widespread use in the nuclear industry and weapons. We proposed a simple and convenient wet-state SERS method for uranyl detection based on the citrate-stabilized silver nanoparticles. The effect of citrate on the detection performance was also discussed. By using the citrate as an internal reference to normalize the peak of uranyl, a quantitative analysis was achieved and a good linear relationship of uranyl concentration from 0.2 to 5 µM with the limit of detection of 60 nM was obtained. With its simplicity, convenience and cost-effectiveness, this method has great potential for the detection of other molecules also.

## 1. Introduction

Uranium (with a high density of 19.2 g cm^−3^), as one of the most important nuclear sources, has shown great application in the nuclear industry and weapons [1,2]. The widespread use of uranium produces a large amount of nuclear wastes which may migrate to the groundwater, bringing long-term health damage to human beings due to its radioactive properties and chemical toxicity [3,4]. Trace amounts of uranium have been found in plants, animals and even in human urine. Hence, real-time monitoring of uranium concentration in ecosystems, environmental waters, and even in the human body is of significance—particularly for nuclear workers.

Uranium exists in the environment usually with two redox states: U(VI) and U(IV). U(IV) is relatively insoluble and can be easily precipitated as uranium dioxide (UO_2_) or can be oxidized to U(VI) [5]. The most common and thermodynamically stable form of U(VI) is uranyl ion (UO_2_^2+^). It is soluble and mobile, which may harm body organs easily and attract more attention by analytical chemists. Traditional instrument-based methods, including mass spectrometry (MS), high-performance liquid chromatography (HPLC), inductively coupled plasma atomic emission spectroscopy (ICP-AES) and X-ray fluorescence spectroscopy [6–8], cannot detect nuclear materials easily. There is a dire need for the development of a simple and portable method for onsite and real-time monitoring of uranium.

Surface-enhanced Raman scattering (SERS) technique [9–13] has the advantages of simplicity, convenience, high sensitivity, non-requirement of pretreatment of analysed sample and has great potential in the rapid and sensitive detection of inorganic ions, organic molecules, even biomacromolecules [14–17]. Many efforts have been made for SERS detection of uranyl ion by fabricating different types of SERS substrates, such as silver-doped sol–gel [18], gold nanostars [19], Al_2_O_3_-coated silver nanorod [20], silver nanoparticles conjugated reduced graphene oxide nanosheets [21] and so on. SERS measurements of those based on ordered nanostructure substrates (dry-state substrate) fabricated by self-assembly or vapour deposition methods usually need drying the sample onto the substrate, which is difficult to obtain quantitative detection due to the coffee-ring effect and substrate-self defect. What is more, the fabrication process of the dry-state substrate is complex and expensive, which shows no commercial competitive advantages.

**Scheme 1.**
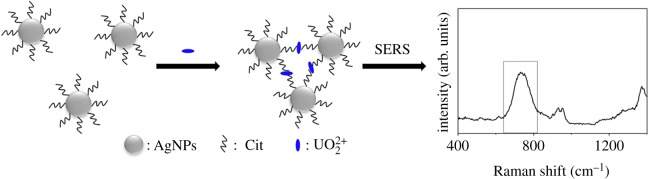
Schematic of the citrate-stabilized AgNPs for SERS detection of UO_2_^2+^.

In this study, we reported a very simple and convenient wet-state method to detect uranyl ion (UO_2_^2+^) by silver colloid by adjusting the amount of citrate to get good sensitivity. Citrate (Cit) has three functions: (1) as the stabilizer to stabilize silver nanoparticles; (2) as the complexing agent to capture uranyl ion through chelation between COO^−^ and UO_2_^2+^ (scheme 1) [22] and (3) as the internal molecule to normalize the Raman signal of uranyl ion to eliminate the adverse effects of the matrix and other external factors. This method is user-friendly and inexpensive and can be achieved in any normal laboratories by any manipulators. What is more, the entire sample fabrication and detection process only needs several seconds if silver colloid had been synthesized previously, which makes it possible for onsite and real-time UO_2_^2+^ monitoring in the environment.

## 2. Material and methods

### 2.1. Chemicals and materials

Uranyl nitrate hexahydrate was obtained from the China National Nuclear Corporation (Lanzhou, China). It was dissolved in ultrapure water to make a 10^−2^ M stock solution, and then diluted to the final concentration before use. Trisodium citrate dihydrate (99%) and silver nitrate (AgNO_3_, ≥99.8%) were purchased from Aladdin. All reagents were used without further purification. Ultrapure water with a resistivity of approximately 18.0 MΩ cm was used throughout the experiments.

### 2.2. Synthesis of silver colloid

The silver colloid was synthesized by the citrate-reduced method according to Lee & Meisel report [23]. In 100 ml of H_2_O, 18 mg of AgNO_3_ was dissolved and then heated to boiling. Two millilitres of 38.8 mM sodium citrate was added with fast stirring. The mixture was kept boiling for about 30 min and was allowed to cool at room temperature under stirring.

### 2.3. SERS sample fabrication

In a typical fabrication process, 8 ml of freshly prepared silver colloid was added into the tube and centrifuged at a speed of 6000 r.p.m. Then 2 ml of water was added to disperse silver nanoparticles (AgNPs) by ultrasonication. The fourfold concentrated AgNP colloid was obtained and used for SERS detection. Citrate cannot be removed completely by this method due to its strong absorption ability on the silver surface. After centrifugation is done, the retained citrate can be absorbed on the surface of AgNPs to protect them from aggregation. For a SERS sample, 10 µl of standard uranyl concentration aqueous solution was added into 90 µl of condensed silver colloid, and the value of Raman spectrum was collected immediately. To characterize the effect of citrate on the intensity of uranyl peak, four silver colloid samples, containing different amounts of citrate, were fabricated and were denoted as A, B, C and D. Eight millilitres of silver colloid was centrifuged and condensed four times by adding: (A) 2 ml of 0.02 wt% trisodium citrate aqueous solution, (B) 2 ml of 0.01 wt% trisodium citrate, (C) 2 ml of water and (D) the colloid was centrifuged and washed with water twice and condensed by adding 2 ml of water. Here, the amount of citrate in sample D with twice centrifugation is lower than that of sample C.

### 2.4. Measurements

The morphology of silver nanoparticles was characterized by a scanning electron microscope (SEM, JEOL-JMS-7001F), the absorption spectra were obtained by a UV–vis spectrometer (SHIMADZU, UV-1800). SERS measurements were conducted on a LabRam Xplora confocal Raman spectrometer (Horiba Jobin Yvon). The laser with the excitation wavelength of 532 nm was used in the experiment, and the laser power is approximately 2.5 mW with the collection time of 10 s. The Raman scattering signal was collected with a numerical aperture (NA) microscopic objective from Olympus (50×, NA = 0.5). For quantitative detection of uranyl ion, five different positions were randomly selected and the SERS intensity was obtained by averaging the relative Raman signals.

## 3. Results

**Figure 1.**
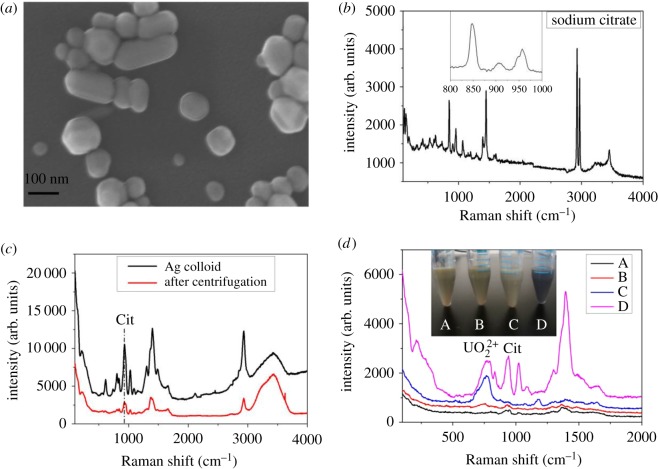
(*a*) SEM image of AgNPs; (*b*) Raman spectrum of sodium citrate crystal; (*c*) Raman spectra of Ag colloid before and after centrifugation; (*d*) SERS of 10 µM UO_2_^2+^ under the condition of A–D. 8 ml of silver colloid was centrifuged and condensed four times by adding: (A) 2 ml of 0.02 wt% trisodium citrate aqueous solution, (B) 2 ml of 0.01 wt% trisodium citrate, (C) 2 ml of water and (D) the colloid was centrifuged and washed with water twice and condensed by adding 2 ml of water.

Compared with gold, AgNPs are superior in terms of SERS performance and cost-effectiveness [24]. With the fast development of nanotechnique, although the shape of the nanostructure is abundant (nanoplates, nanorods, nanoflowers, nanosatellites, etc.) and can be precisely controlled [25–28], nanosphere is the most easily synthesized and also the most stabilized structure. Seen from the point of ease and convenience, silver nanosphere structure (figure 1*a*) synthesized by citrate-reduced method was chosen as the SERS substrate here [23,29]. The low-magnification SEM image (see electronic supplementary material, figure S1) shows that the average size of AgNPs with rather narrow size distribution is approximately 60 nm, which guarantees high sensitivity, because larger-sized nanoparticles can produce a more sensitive signal. The aggregates may be produced from the nanoparticle aggregation when the colloid was drying. Excess citrate can be adsorbed on the surface of silver nanoparticle to form an electric double layer to protect silver from aggregation. However, one important point that many researchers may neglect in their SERS studies is the Raman signal interference of citrate. Figure 1*b* shows the Raman spectrum of solid trisodium citrate; there are five peaks with strong scattering intensities at 848, 957, 1448, 2928, 2969 cm^−1^. Seen from the SERS of silver colloid (figure 1*c*), the three C-COO stretching modes (μ(C-COO)) of Cit exhibit strong Raman signal at 930 cm^−1^ [30], which moves towards lower wavenumbers compared with that of solid citrate at 957 cm^−1^ (figure 1*b*) due to the charge transfer between Cit and Ag. The high background signal will cause an adverse effect on molecule detection, especially during trace analysis. However, after centrifugation, the background signal of Cit decreased markedly (figure 1*c*), thus the amount of citrate in silver colloid for SERS analysis is of significance.

To understand the effect of Cit on the SERS of UO_2_^2+^, a set of control experiments were performed by adjusting the amount of Cit in silver colloid. Four samples were designed: 8 ml of silver colloid was centrifuged and condensed four times by adding: (A) 2 ml of 0.02 wt% trisodium citrate aqueous solution, (B) 2 ml of 0.01 wt% trisodium citrate, (C) 2 ml of water and (D) the colloid was centrifuged and washed with water for two times and condensed by adding 2 ml of water. Centrifugation wash of silver nanoparticles cannot remove citrate fully, thus the amount of citrate retained in colloid in C is larger than that in D. There is a small change in the *λ*_SPR_ of AgNPs from samples A to C (see electronic supplementary material, figure S2) after adding 10 µM of UO_2_^2+^, showing little aggregation of AgNPs. However, the intensity of *λ*_SPR_ in D (see electronic supplementary material, figure S1) decreased remarkably and a new peak at 700 nm appeared, and the silver colloid changed from grey green to dark simultaneously, indicating the severe aggregation of AgNPs. The aggregation of AgNPs brings difficulty for quantitative analysis of uranyl ion. Figure 1*d* shows the SERS spectra of 10 µM of UO_2_^2+^. The relatively broad and asymmetric peak near 750 cm^−1^ is attributed to the *υ*_1_ symmetric stretch of the uranyl ion of uranyl complexes. The ligands have a great effect on the Raman shift of O=U=O [31,32]. Uranyl citrate forms trimeric species with the 3 : 3 and 3 : 2 U : Cit complexes at the near-neutral pH region, exhibiting a complicated complex interaction [22]. The SERS band of uranyl is lower (750 cm^−1^) than uranyl citrate Raman band (832 cm^−1^) (see electronic supplementary material, figure S3), indicating strong coordination between uranyl and silver along with Cit. The strong chemical interactions between uranyl ion and ligands weaken the axial U=O band intensity due to the extensive electron density transfer from citrate and silver surface to the equatorial plane of absorbed uranyl ions, causing the uranyl band to shift from 870 cm^−1^ to a lower wavenumber, which has been reported in the previous literature [33–35]. Sample C obtains a good SERS signal of uranyl (figure 1*d*). The intensity of uranyl band decreases with the increase of citrate (figure 1*d*, A to C). The increase of citrate will protect AgNPs from aggregation and widen the distance between adjacent AgNPs, and thus decrease the Raman intensity. In addition, the excess citrate free in solution will coordinate with the UO_2_^2+^, preventing UO_2_^2+^ from close to AgNPs, which has no contribution to the intensity of the uranyl band (Samples A and B). Proper aggregation can minimize the distance between adjacent AgNPs to create a ‘hot spot’ (sample C), but overage aggregation causes the sedimentation of silver, which weakens the signal of uranyl and brings high background signal, for instance, in the circumstance of sample D (figure 1*d*, C cf. D). So sample C is an appropriate choice for uranyl detection.

**Figure 2.**
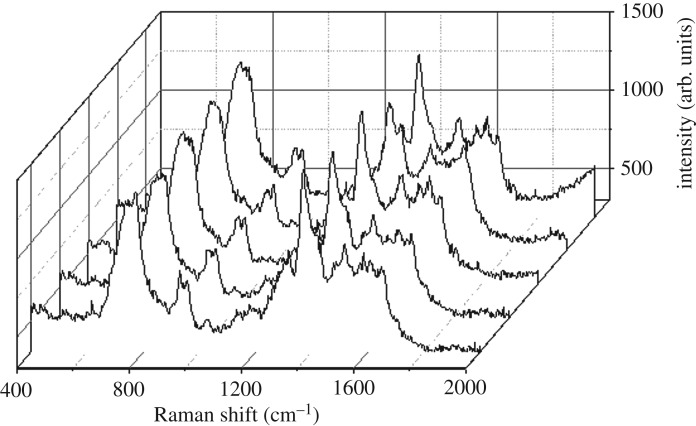
SERS of 5 µM uranyl with five times of continuous measurement under the condition of sample C.

**Figure 3.**
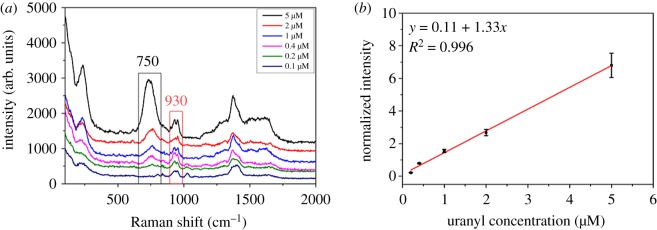
(*a*) SERS of uranyl ion with a concentration from 0.1 to 5 µM. (*b*) The calibration curve of uranyl ion concentration with its relative SERS intensity (*I*_uranyl_*/I*_citrate_) by citrate as the internal reference.

To verify the analytical performance of the proposed wet-state method for uranyl ion, different uranyl ion concentration was determined by adding standard UO_2_^2+^ in sample C. The SERS signal of uranyl species tended to be stable through initial fluctuation, as shown in figure 2, making quantitative detection possible. The environmental factors (temperature, humidity, etc.), the instrumental conditions (laser intensity, focal distance, etc.), sample distinction (colloid concentration, sample cup size, etc.) and human factor will affect the measurement result, which make quantitative detection difficult (see electronic supplementary material, figure S4) [36]. To eliminate the effects of external factors, here we established an internal reference with the peak of the citrate centred at 930 cm^−1^ to normalize the peak of uranyl centred at 750 cm^−1^. Figure 3*a* shows the SERS spectra of uranyl ion with the concentration from 0.1 to 5 µM. After calibration and normalization by citrate, a good linear relationship (*R*^2^ = 0.996) can be obtained between the relative Raman intensity (*I*_uranyl_*/I*_citrate_) and the uranyl concentration from 0.2 to 5 µM (figure 3*b*). The limit of detection (LOD) (60 nM) was calculated by three standard deviations of the blank measurements, showing a good sensitivity. Table 1 lists the comprehensive comparison results of the proposed wet-state method with others reported in the literature [20,34,37–39]. The LOD of our proposed SERS method is higher than those obtained based on DNAzyme or complex nanostructures, while the advantages such as simplicity, rapidness and convenience make it a forceful competitor in real-time environmental monitoring. What is more, the LOD of the proposed strategy is still better than those of photometry and SERS based on modified gold nanoparticles. Although it is difficult to detect the 60 nM of UO_2_^2+^ in the experiment due to the intrinsic spectral background of the blank silver colloid, 200 nM was easily detected in this study.

**Table 1.** An overview for uranyl ion detection.

materials used	method applied	LOD	refs
DNAzyme, AuNPs	colorimetry	50 nM	[37]
gold nanoparticles	photometry	500 nM	[38]
gold nanoparticles	SERS	800 nM	[34]
silver nanorod	SERS	1 nM	[20]
DNAzyme, hairpins	electrochemistry	2 pM	[39]
condensed AgNPs	SERS	60 nM	this paper

**Figure 4.**
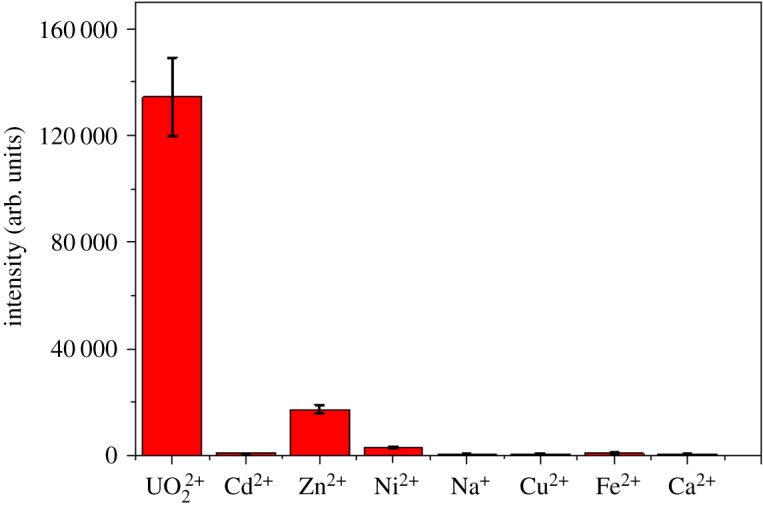
The selectivity of the proposed strategy for UO_2_^2+^: 5 µM of UO_2_^2+^; 500 µM of Na^+^, Zn^2+^, Ca^2+^, Cu^2+^, Fe^2+^, Ni^2+^ and Cd^2+^.

To further evaluate the selectivity of the proposed wet-state SERS strategy for uranyl ion detection, a series of contrast experiments were conducted, including Na^+^, Zn^2+^, Ca^2+^, Cu^2+^, Fe^2+^, Ni^2+^ and Cd^2+^, as shown in figure 4. These impure metal ions did not lead to the signal enhancement. At the same time, the presence of other competitive metal ions had no effect on the relative Raman intensity of uranyl ion (see electronic supplementary material, figure S5), indicating a good sensitivity. The reproducibility of the proposed strategy was evaluated by six repetitive measurements for 1 µM UO_2_^2+^, and the intra-assay relative standard deviation (RSD) was 10% estimated. In addition, the RSD of the inter-assay for UO_2_^2+^ was determined (8.7%) by using five batches of different Ag nanoparticle colloids. These results showed a good selectivity and reproducibility of the proposed method for UO_2_^2+^ detection.

To investigate the validity of the proposed method to environmental sample, a known amount of uranyl ion was added into the tap water sample. These three spiked samples with the uranyl ion concentration of 5, 2 and 1 µM were measured, as shown in table 2. The recoveries and RSDs of three spiked uranyl ion samples in real tap water are from 86% to 105% and 7% to 10%, respectively. These results indicate that our proposed method has great potential for rapid detection of uranyl ion in environmental samples.

**Table 2.** Determination of UO_2_^2+^ in tap water with citrate-stabilized silver nanoparticles.

sample	add (μM)	found (μM)	recovery (%)	RSD (%)
1	5	5.25	105	10.1
2	2	1.85	92.5	8.22
3	1	0.86	86.0	7.23

## 4. Conclusion

We reported a very simple SERS method for rapid detection of uranyl ion based on the citrate-stabilized silver colloid. Owing to the strong interaction between uranyl and citrate as well as silver, uranyl can be captured into the ‘hot spot’ zone, and uranyl ion as low as 200 nM was easily detected by the proposed method, showing an acceptable sensitivity. A good linear plot between the relative intensity and the uranyl concentration was attained by using the residual citrate as the internal reference to normalize the peak of uranyl. Our proposed strategy shows great potential for uranyl real-time and fast analysis in the environment. Furthermore, the internal reference strategy adopted in this paper will push SERS quantitative detection to be more veracious and reliable.

## Supplementary Material

Rapid and sensitive detection of uranyl ion with citrate stabilized silver nanoparticles by surfaced-enhanced Raman scattering technique

## Data accessibility

The data supporting this article have been uploaded as part of the electronic supplementary material.

## References

[1] Kubatko KA, Helean KB, Navrotsky A, Burns PC 2003 Stability of peroxide-containing uranyl minerals. Science 302, 1191–1193. (10.1126/science.1090259) 14615533

[2] Faa A, Gerosa C, Fanni D, Floris G, Eyken PV, Lachowicz JI, Nurchi VM 2018 Depleted uranium and human health. Curr. Med. Chem. 25, 49–64. (10.2174/0929867324666170426102343) 28462701

[3] Biswas B, Mougel V, Pecaut J, Mazzanti M 2011 Base-driven assembly of large uranium oxo/hydroxo clusters. Angew. Chem. Int. Ed. Engl. 50, 5745–5748. (10.1002/anie.201101327) 21567695

[4] Maher K, Bargar JR, Brown GE Jr 2013 Environmental speciation of actinides. Inorg. Chem. 52, 3510–3532. (10.1021/ic301686d) 23137032

[5] Peled Y, Krent E, Tal N, Tobias H, Mandler D 2015 Electrochemical determination of low levels of uranyl by a vibrating gold microelectrode. Anal. Chem. 87, 768–776. (10.1021/ac503719r) 25437433

[6] Dejeant A, Bourva L, Sia R, Galoisy L, Calas G, Phrommavanh V, Descostes M 2014 Field analyses of 238 U and 226 Ra in two uranium mill tailings piles from Niger using portable HPGe detector. J. Environ. Radioact. 137, 105–112. (10.1016/j.jenvrad.2014.06.012) 25036918

[7] Jaison P, Telmore VM, Kumar P, Aggarwal SK 2011 Determination of uranium in seawater samples by liquid chromatography using mandelic acid as a complexing agent. J. Chromatogr. Sci. 49, 657–664. (10.1093/chrsci/49.9.657) 22586240

[8] Xu M, Frelon S, Simon O, Lobinski R, Mounicou S 2014 Development of a non-denaturing 2D gel electrophoresis protocol for screening *in vivo* uranium-protein targets in *Procambarus clarkii* with laser ablation ICP MS followed by protein identification by HPLC–Orbitrap MS. Talanta 128, 187–195. (10.1016/j.talanta.2014.04.065) 25059147

[9] Schlucker S 2014 Surface-enhanced Raman spectroscopy: concepts and chemical applications. Angew. Chem. Int. Ed. Engl. 53, 4756–4795. (10.1002/anie.201205748) 24711218

[10] Jiang J, Zou S, Ma L, Wang S, Liao J, Zhang Z 2018 Surface-enhanced Raman scattering detection of pesticide residues using transparent adhesive tapes and coated silver nanorods. ACS Appl. Mater. Interfaces 10, 9129–9135. (10.1021/acsami.7b18039) 29470045

[11] Yang N, You TT, Liang X, Zhang CM, Jiang L, Yin PG 2017 An ultrasensitive near-infrared satellite SERS sensor: DNA self-assembled gold nanorod/nanospheres structure. RSC Adv. 7, 9321–9327. (10.1039/C6RA27185E)

[12] Chen S, Dong L, Yan M, Dai Z, Sun C, Li X 2018 Rapid and sensitive biomarker detection using molecular imprinting polymer hydrogel and surface-enhanced Raman scattering. R. Soc. open sci. 5, 171488 (10.1098/rsos.171488) 29410851 PMC5792928

[13] Wang Y, Yan B, Chen L 2013 SERS tags: novel optical nanoprobes for bioanalysis. Chem. Rev. 113, 1391–1428. (10.1021/cr300120g) 23273312

[14] Mosier-Boss PA 2017 Review of SERS substrates for chemical sensing. Nanomaterials 7, 142–172. (10.3390/nano7060142) PMC5485789 28594385

[15] Li J, Chen L, Lou T, Wang Y 2011 Highly sensitive SERS detection of As^3+^ ions in aqueous media using glutathione functionalized silver nanoparticles. ACS Appl. Mater. Interfaces 3, 3936–3941. (10.1021/am200810x) 21916441

[16] Ding Y, Wang S, Li J, Chen L 2016 Nanomaterial-based optical sensors for mercury ions. Trac-trends Anal. Chem. 82, 175–190. (10.1016/j.trac.2016.05.015)

[17] Chen L, Qi N, Wang X, Chen L, You H, Li J 2014 Ultrasensitive surface-enhanced Raman scattering nanosensor for mercury ion detection based on functionalized silver nanoparticles. RSC Adv. 4, 15 055–15 060. (10.1039/C3RA47492E)

[18] Bao L, Mahurin S, Haire R, Dai S 2003 Silver-doped sol−gel film as a surface-enhanced Raman scattering substrate for detection of uranyl and neptunyl ions. Anal. Chem. 75, 6614–6620. (10.1021/ac034791+) 16465717

[19] Lu G, Johns AJ, Neupane B, Phan HT, Cwiertny DM, Forbes TZ, Haes AJN 2018 Matrix-independent surface-enhanced Raman scattering detection of uranyl using electrospun amidoximated polyacrylonitrile mats and gold nanostars. Anal. Chem. 90, 6766–6772. (10.1021/acs.analchem.8b00655) 29741873 PMC6354247

[20] Jiang J et al. 2017 SERS detection and characterization of uranyl ion sorption on silver nanorods wrapped with Al_2_O_3_ layers. Microchim. Acta 184, 2775–2782. (10.1007/s00604-017-2286-0)

[21] Dutta S, Ray C, Sarkar S, Pradhan M, Negishi Y, Pal T 2013 Silver nanoparticle decorated reduced graphene oxide (rGO) nanosheet: a platform for SERS based low-level detection of uranyl ion. ACS Appl. Mater. Interfaces 5, 8724–8732. (10.1021/am4025017) 23947790

[22] Basile M, Unruh D, Gojdas K, Flores E, Streicher L, Forbes T 2015 Chemical controls on uranyl citrate speciation and the self-assembly of nanoscale macrocycles and sandwich complexes in aqueous solutions. Chem. Commun. 51, 5306–5309. (10.1039/C4CC08657K) 25469487

[23] Lee PC, Meisel D 1982 Adsorption and surface-enhanced Raman of dyes on silver and gold sols. J. Phys. Chem. B 86, 3391–3395. (10.1021/j100214a025)

[24] Gadogbe M, Ansar SM, Chu IW, Zou S, Zhang D 2014 Comparative study of the self-assembly of gold and silver nanoparticles onto thiophene oil. Langmuir 30, 11 520–11 527. (10.1021/la502574p) 25198286

[25] Han C, Yao Y, Wang W, Qu L, Bradley L, Sun S, Zhao Y 2017 Rapid and sensitive detection of sodium saccharin in soft drinks by silver nanorod array SERS substrates. Sens. Actuators B 251, 272–279. (10.1016/j.snb.2017.05.051)

[26] Powell JA, Venkatakrishnan K, Tan B 2017 A primary SERS-active interconnected Si-nanocore network for biomolecule detection with plasmonic nanosatellites as a secondary boosting mechanism. RSC Adv. 7, 33 688–33 700. (10.1039/C7RA01970J)

[27] Khan FA, Ajmal CM, Bae S, Seo S, Moon H, Baik S 2018 Silver nanoflower decorated graphene oxide sponges for highly sensitive variable stiffness stress sensors. Small 14, e1800549 (10.1002/smll.201800549) 29756315

[28] Zhang XY, Hu A, Zhang T, Lei W, Xue XJ, Zhou Y, Duley WW 2011 Self-assembly of large-scale and ultrathin silver nanoplate films with tunable plasmon resonance properties. ACS Nano 5, 9082–9092. (10.1021/nn203336m) 21955107

[29] Jiang J, Wang S, Wu H, Zhang J, Li H, Jia J, Wang X, Liao J 2015 Facile and rapid fabrication of large-scale silver nanoparticles arrays with high SERS performance. RSC Adv. 5, 105 820–105 824. (10.1039/C5RA22358J)

[30] Munro CH, Smith WE, Garner M, Clarkson J, White PC 1995 Characterization of the surface of a citrate-reduced colloid optimized for use as a substrate for surface-enhanced resonance Raman scattering. Langmuir 11, 3712–3720. (10.1021/la00010a021)

[31] Nguyentrung C, Begun GM, Palmer DA 1992 Aqueous uranium complexes: Raman-spectroscopic study of the complex-formation of the diosouranium (VI) ion with a variety of inorganic and organic-ligands. Inorg. Chem. 31, 5280–5287. (10.1021/ic00051a021)

[32] Rowland CE, Kanatzidis MG, Soderholm L 2012 Tetraalkylammonium uranyl isothiocyanates. Inorg. Chem. 51, 11 798–11 804. (10.1021/ic301741u) 23072277

[33] Wang S, Jiang J, Wu H, Jia J, Shao L, Tang H, Ren Y, Chu M, Wang X 2017 Self-assembly of silver nanoparticles as high active surface-enhanced Raman scattering substrate for rapid and trace analysis of uranyl (VI) ions. Spectrochim. Acta, Part A 180, 23–28. (10.1016/j.saa.2017.02.042) 28262580

[34] Ruan C, Luo W, Wang W, Gu B 2007 Surface-enhanced Raman spectroscopy for uranium detection and analysis in environmental samples. Anal. Chim. Acta 605, 80–86. (10.1016/j.aca.2007.10.024) 18022414

[35] Bhandari D, Wells SM, Retterer ST, Sepaniak MJ 2009 Characterization and detection of uranyl ion sorption on silver surfaces using surface enhanced Raman spectroscopy. Anal. Chem. 81, 8061–8067. (10.1021/ac901266f) 19737007

[36] Li R, Yang G, Yang J, Han J, Liu J, Huang M 2016 Determination of melamine in milk using surface plasma effect of aggregated Au@SiO_2_ nanoparticles by SERS technique. Food Control 68, 14–19. (10.1016/j.foodcont.2016.03.009)

[37] Lee JH, Wang Z, Liu J, Lu Y 2008 Highly sensitive and selective colorimetric sensors for uranyl (UO_2_^2+^): Development and comparison of labeled and label-free DNAzyme-gold nanoparticle systems. J. Am. Chem. Soc. 130, 14 217–14 226. (10.1021/ja803607z) 18837498 PMC2667950

[38] Liang Y, He Y 2016 Arsenazo III-functionalized gold nanoparticles for photometric determination of uranyl ion. Microchim. Acta 183, 407–413. (10.1007/s00604-015-1659-5)

[39] Yun W, Cai D, Jiang J, Wang X, Liao J, Zhang P, Sang G 2016 An ultrasensitive electrochemical biosensor for uranyl detection based on DNAzyme and target-catalyzed hairpin assembly. Microchim. Acta 183, 1425–1432. (10.1007/s00604-016-1778-7)

