# Supplementary material for: Rapid and sensitive detection of uranyl ion with citrate-stabilized silver nanoparticles by the surface-enhanced Raman scattering technique
Source: R Soc Open Sci. 2018 Nov 28;5(11):181099. doi: 10.1098/rsos.181099 (PMC6281930; doi:10.1098/rsos.181099)
Supplement: Rapid and sensitive detection of uranyl ion with citrate stabilized silver nanoparticles by surfaced-enhanced Raman scattering technique [file rsos181099supp1.docx]

Electronic Supplementary Material for:

**Rapid and sensitive detection of uranyl ion with citrate stabilized silver nanoparticles by surfaced-enhanced Raman scattering technique†**

Jiaolai Jiang,^a^ Shaofei Wang,^a^ Hui Deng,^a^ Haoxi Wu,^a^ Jun Chen,*^a^ and Junsheng Liao*^a^

^a^ Institute of Materials, China Academy of Engineering Physics, P. O. Box No.9-11, Mianyang, Sichuan, 621907, P. R. China.

E-mail: junchenspc@caep.cn, [jshliao711@163.com](mailto:jshliao711@163.com).

This PDF file include:

Figure. S1-S5


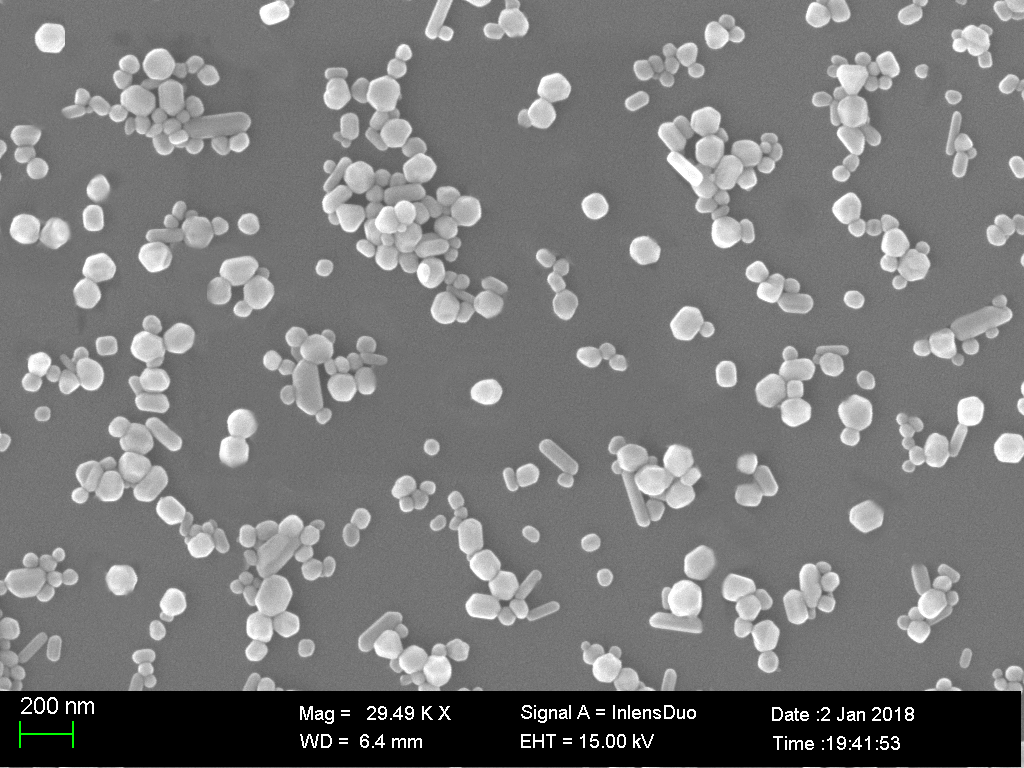


Figure S1. The SEM image of AgNPs dispersed on Si.


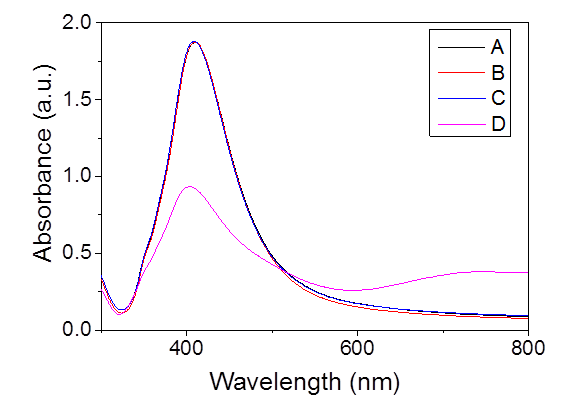


Figure S2. The UV-vis spectra of silver colloid samples A to D. The 8 mL colloids were condensed 4 times through centrifugation by adding: A) 2 mL of 0.02 wt% trisodium citrate aqueous solution, B) 2 mL of 0.01 wt% trisodium citrate, C) 2 mL of water, and D) 2 mL of water after twice centrifugation, respectively.


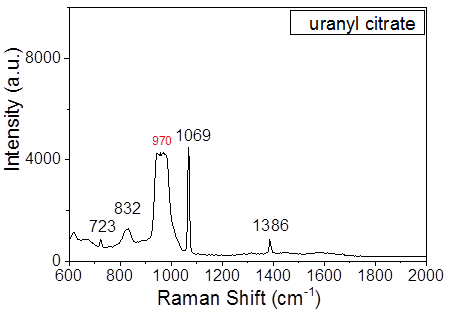


Figure S3. The Raman spectrum of 0.01 M uranyl citrate on Si. The 970 cm^-1^ is attributed to the band of Si.


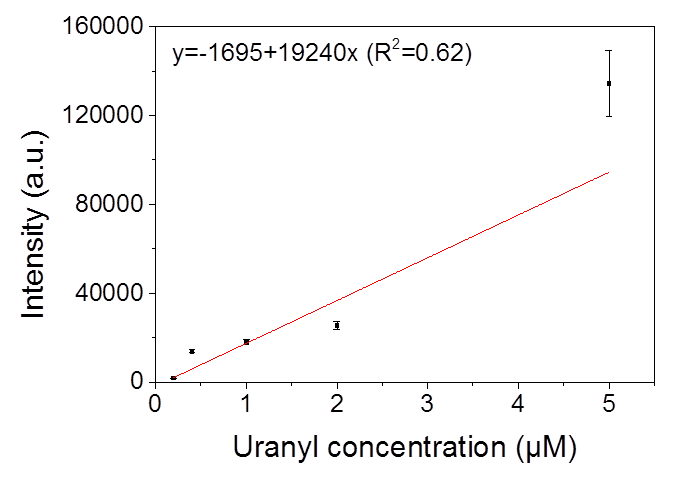


Figure S4. Calibration plot of uranyl concentration with SERS intensity, which shows poor liner relationship between them.


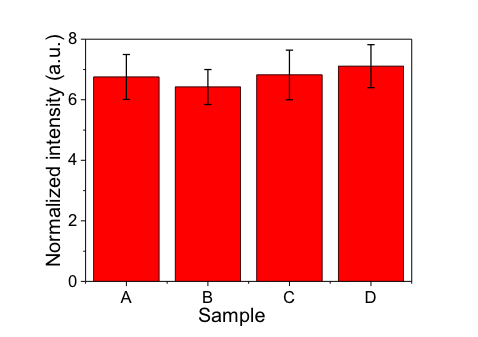


Figure S5. The selectivity of the proposed strategy for UO_2_^2+^ in the presence of competitive metal ions: (A) 5 μM of UO_2_^2+^; (B) 5 μM of UO_2_^2+^ containing 100 μM of Na^+^; (C) 5 μM of UO_2_^2+^ containing 100 μM of Na^+^ and Ca^2+^; (D) 5 μM of UO_2_^2+^ containing 100 μM of Na^+^, Ca^2+^ and Cu^2+^.
